# Bacteria associated with the cereal leaf beetle act as the insect’s allies in adapting to protease inhibitors, but impair its development in laboratory condition

**DOI:** 10.1038/s41598-025-23674-9

**Published:** 2025-11-14

**Authors:** Beata Wielkopolan, Alicja Szabelska-Beręsewicz, Aleksandra Obrępalska-Stęplowska

**Affiliations:** 1 Department of Monitoring and Signaling of Agrophages, Institute of Plant Protection–National Research Institute, Poznan, Poland; 2 Department of Mathematical and Statistical Methods, Poznań University of Life Sciences, Poznan, Poland; 3 Department of Molecular Biology and Biotechnology, Institute of Plant Protection–National Research Institute, Poznan, Poland

**Keywords:** Cereal leaf beetle, Bacteria, Microbiome, Protease inhibitors, Insect development, Insect-bacteria interaction, Ecology, Ecology, Microbiology, Zoology

## Abstract

*Oulema melanopus* [L.] (cereal leaf beetle, CLB) is one of the most serious cereal pests. Plant protease inhibitors (PIs) are known for their insecticidal properties. The role of CLB-associated bacteria in insect adaptation to PIs is not yet known. We investigated the role of CLB-associated bacteria in adaptation to PIs, and whether the reduction of bacteria will affect the CLB development. We found a decrease in proteases activity in insects with a diminished bacterial community compared to those with an intact bacterial community. Thus, the study showed that the CLB-associated bacteria participate in the adaptation of CLB larvae to PIs. On the other hand, regardless of the type of PI used, ultimately a higher survival rates were recorded for larvae with a reduced bacterial community compared to insects with a natural microbiome in laboratory conditions. In such conditions, higher larval survival rates and a higher percentage of larvae reaching the pupal and imago stages were recorded in insects whose bacterial community was reduced. Since the CLB bacterial microbiome showed a negative impact on the development of the insect’s host and its survival in response to PIs in laboratory conditions, it can be concluded that CLB-associated bacteria can be an ally of its insect host, but also an adversary when conditions are not optimal for symbiosis.

**Supplementary Information:**

The online version contains supplementary material available at 10.1038/s41598-025-23674-9.

## Introduction

*Oulema melanopus* [L.] (cereal leaf beetle, CLB, Chrysomelidae, Coleoptera) is one of the most serious cereal pests affecting, among others, barley (*Hordeum vulgare* L.), wheat (*Triticum aestivum* L. subsp. *aestivum*) and oats (*Avena sativa* L.)^1,2^. The management of CLB larvae (the main harmful stage) in cereal crops relies heavily on chemical protection^1,3^, the use of which is associated with the disruption of natural ecosystems, the emergence of insecticide-resistant insects, and human health problems^3,4^. The insecticide resistance in some CLB populations was reported^5^.

Plants have evolved a number of defense mechanisms against herbivorous insects, including the synthesis of protease inhibitors (PIs), known for their insecticidal properties. Plant PIs can interfere with insect digestive proteases, disrupting protein digestion and nutritional balance^6–8^, causing impaired growth and development of herbivores^6,9^, making them more vulnerable to their predators, which may enhance the effectiveness of the pest management strategies^9^. PIs may also affect insect survival^6,8,10^. Many studies have examined the potential use of PIs against several economically important pests of agricultural crops^6,8,11–13^, but their effectiveness has been hindered by the insect pests’ adaptation to them^3,14–17^.

As holobionts, insects are associated with diverse microbiota (including Bacteria, Archaea, eucaryotic microorganisms, and viruses) that affect their fitness (positively or negatively)^18,19^. Insect-associated bacteria influence the physiology of their insects host in many ways^20–23^, including helping to overcome dietary barriers^6,24–26^ by supporting insect metabolism and digestive physiology^27^. Since bacteria can influence insect-plant interactions^23^ and participate in the detoxification of harmful substances of plant or synthetic origin, the symbiotic relationship between an insect and its bacteria makes insect control more difficult^4^. In previous research, we revealed, among others, that CLB-associated bacteria mitigated numerous anti-herbivore processes and pathways associated with the synthesis of metabolites and proteins (including PIs) potentially harmful to the insects^14,28^ and that CLB larvae have evolved mechanisms to hamper the deleterious effects of PIs^15^.

In this study, we have undertaken to address the following questions: whether the CLB-associated bacteria are involved in insect adaptation to PIs, and whether bacterial reduction will affect the development of the CLB (reaching the pupal and then adult stage). We assumed that: (i) reduction of CLB larvae-associated bacteria by antibiotic treatment may result in increased larval mortality in response to PIs treatment, (ii) CLB-associated bacteria participate in insect adaptation to the PIs, and (iii) bacterial reduction in CLB larvae will disrupt the digestive processes and, in the consequence, will have negative impact on the development of the insect.

To look for alternative, environmentally friendly CLB management strategies, it is necessary to understand CLB-host plant interactions on molecular and physiological levels, with consideration of the role of CLB-associated bacteria, which can help insects adapt to PIs and resist their effect. Understanding the tripartite relationship between plants, insects, and their microbial communities may facilitat the development of effective, integrated pest management strategies.

## Results

**Fig. 1:**
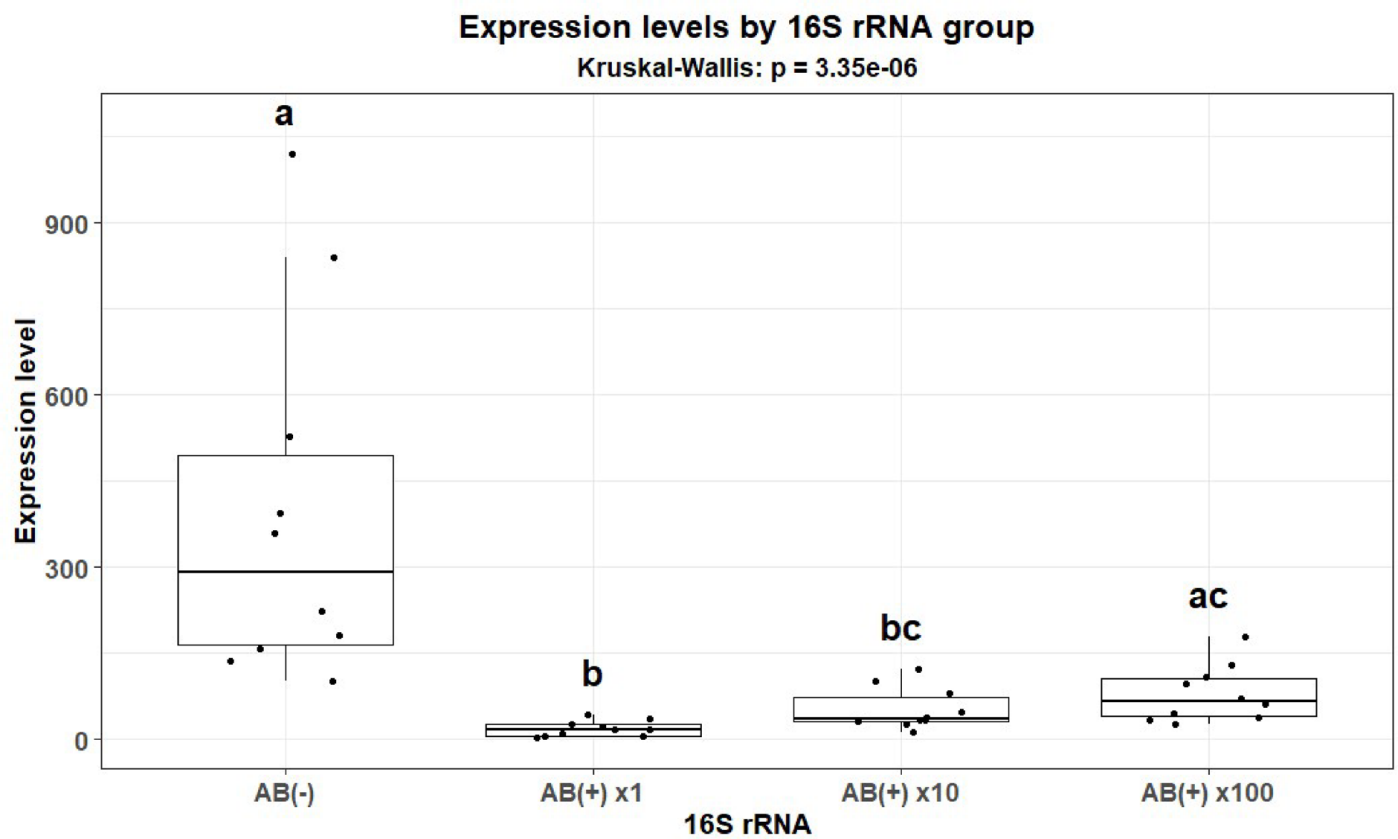
The expression level of the 16S rRNA gene of bacteria associated with CLB larvae. The expression level of the 16S rRNA gene by ddPCR was determined in larvae with a natural microbiome (AB(−)) and in larvae in which the bacterial content was reduced by using the initial antibiotics concentration (AB( +) × 1) and its tenfold (AB( +) × 10) and 100-fold (AB( +) × 100) dilutions. There were 10 CLB larvae for each treatment. Significant codes for *p*-value: *p < 0.05, **p < 0.01, ***p < 0.001.

A significant decrease in the number of bacteria associated with CLB larvae after antibiotic (AB) treatment was observed (as counted by the expression levels of the 16S rRNA of bacteria associated with CLB using digital droplet PCR (ddPCR)). The highest decrease (18-fold compared to AB-untreated insects (insects with a natural microbiome, control)) in the number of bacteria was observed when CLB larvae were treated with the initial (the highest) concentration of AB in comparison to its tenfold and 100-fold dilutions. Therefore, the initial concentration of AB was then used to prepare CLB larvae with a diminished bacterial community for further experiments (Fig. 1 and see Supplementary Fig. S1 online).

### Bacteria associated with CLB larvae have a negative effect on the survival of the insect host exposed to PIs in laboratory conditions

**Fig. 2:**
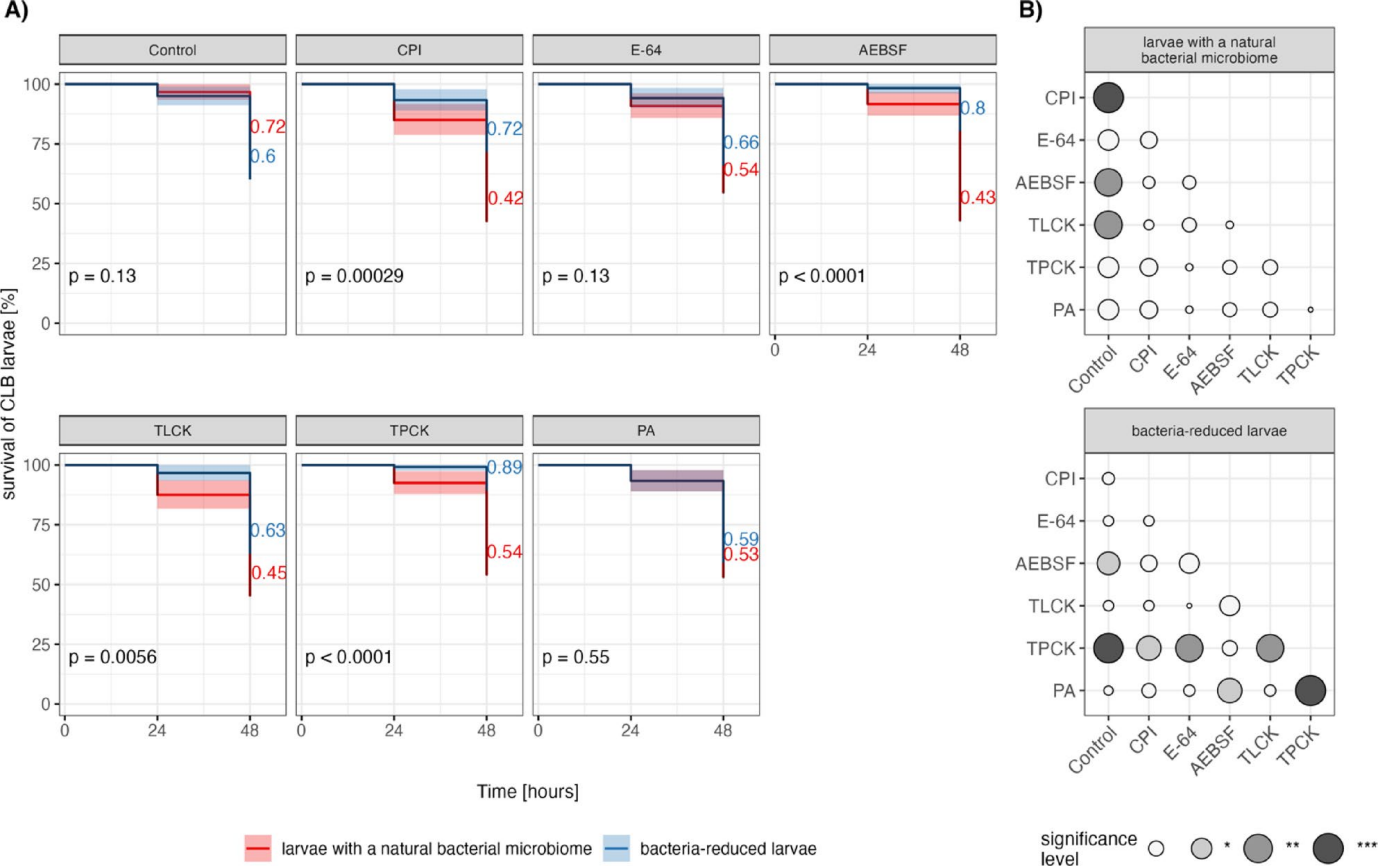
(**A)** Kaplan–Meier curve depicting the survival of CLB larvae [%] with a natural (red lines) and reduced (blue lines) bacterial microbiome exposed to protease inhibitor (CPI, E-64, AEBSF, TLCK, TPCK, PA). Controls were insects with a natural microbiome (neither antibiotics nor protease inhibitor-treated) and larvae with reduced bacterial community (treated only with antibiotics). The experiment involved a total of 12 treatments, with 20 larvae for each. The CLB larvae were collected from wheat fields in Słupia Wielka in 2021 and 2022 and from Pętkowo in 2022. Evaluation of larval survival was performed 24 and 48 h after larvae were exposed to protease inhibitor. Numerical values at the end of survival curves indicate final survival percentages for each treatment group. P-values present in each panel represent significance of statistical comparisons between survival curves performed using log-rank tests. (**B**) Dot plot matrix showing statistically significant pairwise comparisons calculated with log-rank tests between protease inhibitor treatments for larvae with a natural bacterial microbiome (top panel) and larvae with reduced bacterial community (bottom panel). The size and color of the filled circles indicate the level of significance (p-values: *p < 0.05, **p < 0.01, ***p < 0.001). Empty circles indicate non-significant differences. CPI-cocktail of protease inhibitors, E-64-cysteine protease inhibitor, AEBSF-serine protease inhibitor, TLCK-trypsin protease inhibitor, TPCK-chymotrypsin protease inhibitor, PA-aspartyl protease inhibitor.

Firstly, we compared the data from PI-treated larvae with intact (AB-untreated) and reduced bacterial community (AB-treated) to the corresponding controls using Kaplan–Meier survival analysis with log-rank tests for statistical comparisons, i.e., neither AB nor PI-treated larvae and AB-treated only, respectively. In the experiment, 5 types of PIs were used: trans-epoxysuccinyl-L-leucylamido-4 guanidino butane (E-64, cysteine protease inhibitor), pepstatin A (PA, aspartyl protease inhibitor), 4-(2-aminoethyl) benzenesulfonyl fluoride hydrochloride (AEBSF, serine protease inhibitor), Nα-tosyl-L-lysine chloromethyl ketone hydrochloride (TLCK, trypsin protease inhibitor), N-p-tosyl-L-phenylalanine chloromethyl ketone (TPCK, chymotrypsin protease inhibitor), and a cocktail of protease inhibitors (CPI). In groups of larvae with a natural bacterial microbiome, lower larval survival rates were observed regardless of the type of PI used, compared to the control (neither AB nor PI-treated insects) (Fig. 2A), but statistically significant differences in survival rates between PI-treated larvae and control were recorded in response to CPI (log-rank test: χ^2^ = 17.366, df = 1, *p* < 0.001), AEBSF (χ^2^ = 12.574, df = 1, *p* < 0.01) and TLCK (χ^2^ = 13.685, df = 1, *p* < 0.01) (Fig. 2B). In turn, in groups of insects with reduced bacterial community, after the application of CPI, AEBSF and TPCK, the survival rate was higher compared to the control (AB-treated larvae only) (Fig. 2A). But statistically significant differences in larval survival rate between larvae with reduced bacterial community and controls were noted in response to AEBSF (χ^2^ = 7.055, df = 1, *p* < 0.05) and TPCK (χ^2^ = 15.489, df = 1, *p* < 0.001) (Fig. 2B).

Next, we compared the survival rate of larvae with a diminished bacterial community and insects with a natural microbiome, depending on the type of PIs used. Contrary to our assumption, the laboratory assay showed that regardless of the type of PI used, higher survival rates were recorded for larvae with reduced bacterial community compared to insects with a natural bacterial microbiome. Statistically significant differences in survival rate between insects with a natural and reduced bacterial microbiomes were recorded when using CPI (χ^2^ = 13.156, df = 1, *p* < 0.001), AEBSF (χ^2^ = 21.415, df = 1, *p* < 0.001), TPCK (χ^2^ = 22.220, df = 1, *p* < 0.05) and TLCK (χ^2^ = 7.683, df = 1, *p* < 0.01) (Fig. 2A).

Interestingly, in the group of insects that constitute the control, a higher survival rate was noted for insects with a natural microbiome (AB-untreated) than those with a reduced one (AB-treated). But the difference in survival rate is not statistically significant (Fig. 2A).

### Bacteria associated with CLB larvae have a positive impact on protease activity after PI treatment

The next step was to test whether bacteria associated with CLB larvae are involved in the adaptation of their insect host to the PIs, therefore, the total activity (expressed as median fluorescence intensity, MFI) of CLB larvae-associated proteases (that is derived from all CLB larvae tissues) was measured spectrophotometrically in insects depending on the presence of a natural or reduced bacterial microbiome. In the experiment, 5 types of PIs were used: E-64, PA, AEBSF, TLCK, TPCK, and CPI. Protease activity was determined at three pH (4.0, 6.2, 7.6). We indicated that PI treatment, the pH, and reduction of CLB-associated bacteria (AB) have a statistically significant effect on total protease activity (at the *p* < 0.001 level) (Table 1). Almost all interactions between the variables were also statistically significant (*p* < 0.001 for PI x AB, PI x pH, PI x AB x pH) (Table 1).

**Table 1:** ANOVA table for the GLM NB model analyzing protease activity in CLB larvae. The dependent variable was median fluorescence intensity (MFI) representing protease activity levels. Model formula: MFI ~ PI × AB × pH, where PI = protease inhibitor type, AB = reduction of CLB-associated bacteria, pH = buffer pH level. The significance of each variable and the included interactions between them is presented as the *p*-values from chi-square tests. Significant codes for *p*-value: *p < 0.05, **p < 0.01, ***p < 0.001.

	Degrees of freedom	Deviance	*p*-value
PI	6	41.44	< 0.00001***
AB	1	62.40	< 0.00001***
pH	2	39.09	< 0.00001***
PI x ABs	6	32.94	0.00001***
PI x pH	12	55.91	< 0.00001***
AB x pH	2	4.84	0.08892
PI x AB x pH	12	58.41	< 0.00001***

Model specifications:

Model formula: MFI ~ PI × AB × pH.

Distribution family: Negative binomial.

Residual deviance: 126.8 on 84 degrees of freedom.

AIC: 3557.

Overdispersion parameter: 26.54.

Nagelkerke’s R^2^: 0.934.

**Fig. 3:**
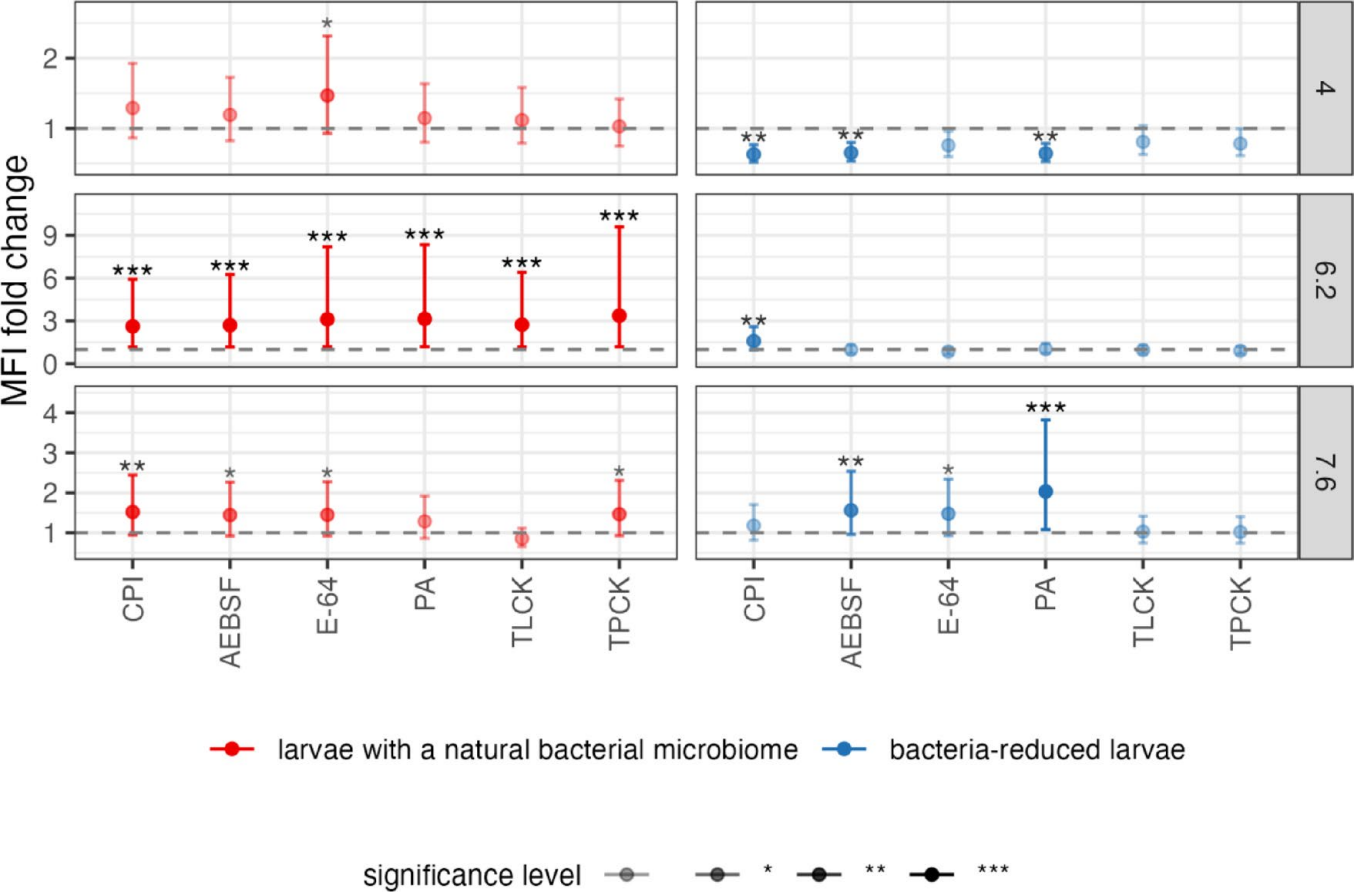
Protease activity fold changes in response to protease inhibitors across different pH conditions. The estimated fold change in median fluorescence intensity value (MFI) of protease inhibitor-treated larvae vs control for each pH value considered (4.0, 6.2, 7.6) is reported. Points represent mean fold changes with 95% confidence intervals (error bars). The data from protease inhibitor-treated insects with a natural and reduced bacterial microbiomes were compared to the corresponding control, i.e., neither antibiotics nor protease inhibitor-treated and treated only with antibiotics, respectively. The experiment involved a total of 12 treatments, with 9 larvae (3 biological replicates of 3 pooled larvae each) for each. The CLB larvae were collected from wheat fields in Słupia Wielka in 2021. Statistical significance was determined using estimated marginal means from GLM negative binomial model with z-tests. The red error bars refer to larvae with a natural bacterial microbiome, the blue error bars refer to insects with reduced bacterial community, dash lines at y = 1 in panels indicated no change from control (neither antibiotics nor protease inhibitor-treated and treated only with antibiotics, respectively). CPI-cocktail of protease inhibitors, AEBSF-serine protease inhibitor, E-64-cysteine protease inhibitor, TLCK-trypsin protease inhibitor, TPCK-chymotrypsin protease inhibitor, PA-aspartyl protease inhibitor. Significant codes for p-value: *p < 0.05, **p < 0.01, ***p < 0.001.

When we compared the MFI value of PI-treated insects with a natural microbiome to control (neither AB nor PI-treated insects), an increase in total protease activity (expressed as MFI) was noted regardless of the PI type and pH value (except for TLCK at pH 7.6). But the most statistically significant increase in MFI value between PI-treated larvae with a natural microbiome and control was recorded for pH 6.2 (z-score: 7.662, *p* < 0.001) (Fig. 3 and see Supplementary Table S1 online). Then, we compared the MFI value of PI-treated CLB larvae with reduced bacterial community to the control (AB-treated larvae only) (Fig. 3 and see Supplementary Table S1 online). Here, noted the opposite effect, namely a decrease in proteolytic activity in PI-treated larvae with reduced bacterial community at pH 4.0 and 6.2 (except for PA and CPI), and an increase at pH 7.6, compared to the control (AB-treated only) (Fig. 3 and see Supplementary Table S1 online). The most statistically significant decrease in MFI value was recorded for CPI (z-score: − 2.905, *p* < 0.01), AEBSF (z-score: -2.687, *p* < 0.01) and PA (z-score: − 2.775, *p* < 0.01) at pH 4.0 and statistically significant increase for CPI (z-score: 2.914, *p* < 0.01) at pH 6.2 and for PA (z-score: 4.474, *p* < 0.001), AEBSF (z-score: 2.811, *p* < 0.01) and E-64 (z-score: 2.462, *p* < 0.05) at pH 7.6 (Fig. 3 and see Supplementary Table S1 online).

The results indicate that CLB larvae with a natural microbiome have developed adaptive mechanisms against PIs manifested by an increase in proteolytic activity. In turn, reduction of bacteria in CLB larvae impairs the insect’s response to PIs, as a decrease in protease activity was observed for pH 4.0 and 6.2 (except for CPI and PA for pH 6.2).

**Fig. 4:**
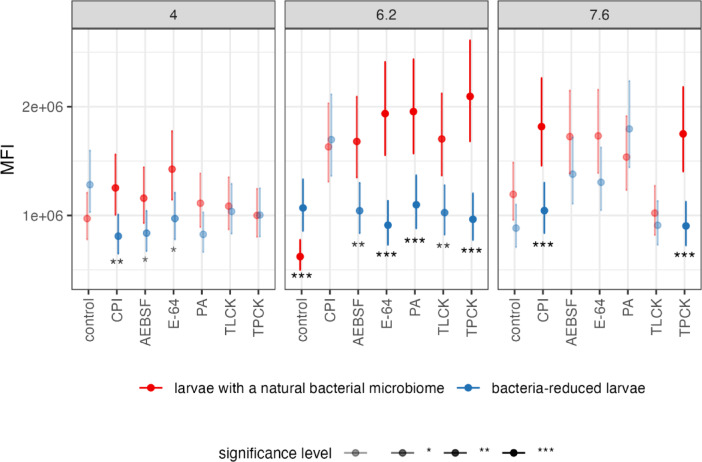
Protease activity profiles across experimental conditions. Impact of pH value (4.0, 6.2 and 7.6), and type of protease inhibitor (CPI, AEBSF, E-64, PA, TLCK, TPCK) on total activity of proteases (represented with median fluorescence intensity (MFI)) of CLB larvae with a natural microbiome and reduced bacterial community, respectively. Controls were insects with a natural microbiome (neither antibiotics nor protease inhibitor treated) and larvae with reduced bacterial community (treated only with antibiotics). The experiment involved a total of 12 treatments, with 9 larvae (3 biological replicates of 3 pooled larvae each) for each, were collected from wheat fields in Słupia Wielka in 2021. Results for each treatment are reported as mean MFI values (dots) together with 95% confidence intervals (whiskers) of means for each combination of variables. Statistical comparisons were performed using GLM NB models with Tukey’s post-hoc correction for multiple comparisons. The red error bars refer to larvae with a natural bacterial microbiome, blue error bars refer to insects with reduced bacterial community. CPI-cocktail of protease inhibitors, AEBSF-serine protease inhibitor, E-64-cysteine protease inhibitor, PA-aspartyl protease inhibitor, TLCK-trypsin protease inhibitor, TPCK-chymotrypsin protease inhibitor. Significant codes for p-value: *p < 0.05, **p < 0.01, ***p < 0.001.

Next, we compared the MFI value of larvae with diminished bacterial community *vs* insects with a natural microbiome, depending on the type of PI and pH value. We assumed that proteolytic activity would be higher in insects with a natural microbiome compared to those with a reduced one in response to PI treatment. Such a result could suggest that bacteria associated with CLB larvae participate in the adaptation of their insect host to PIs. The results confirmed our hypothesis, as after the reduction of bacteria with AB, then after the application of PI, lower protease activity was observed at each of the pH values tested (particularly at pH 6.2) compared to insects with a natural microbiome (except for TPCK at pH 4.0, CPI at pH 6.2 and PA at pH 7.6) (Fig. 4 and see Supplementary Table S2 online). A statistically significant decrease in MFI value in larvae with reduced bacterial community was recorded for pH 6.2 after the application of E-64, PA, TPCK (*p* < 0.001), AEBSF, and TLCK (*p* < 0.01), compared to larvae with a natural microbiome (Fig. 4 and see Supplementary Table S2 online). The greatest decrease in proteolytic activity was observed in larvae with reduced bacterial microbiome after treatment with E-64 (for all three pH values), CPI (at pH 4.0 and 7.6) and TPCK (at pH 6.2 and 7.6) compared to insects with a natural microbiome (Fig. 4 and see Supplementary Table S2 online).

This indicates that reduction of bacteria in the CLB larvae has a negative impact on protease activity after PI treatment (especially those active at pH 6.2), and this mainly involves cysteine and chymotrypsin proteases (Fig. 4 and see Supplementary Table S2 online).

**Fig. 5:**
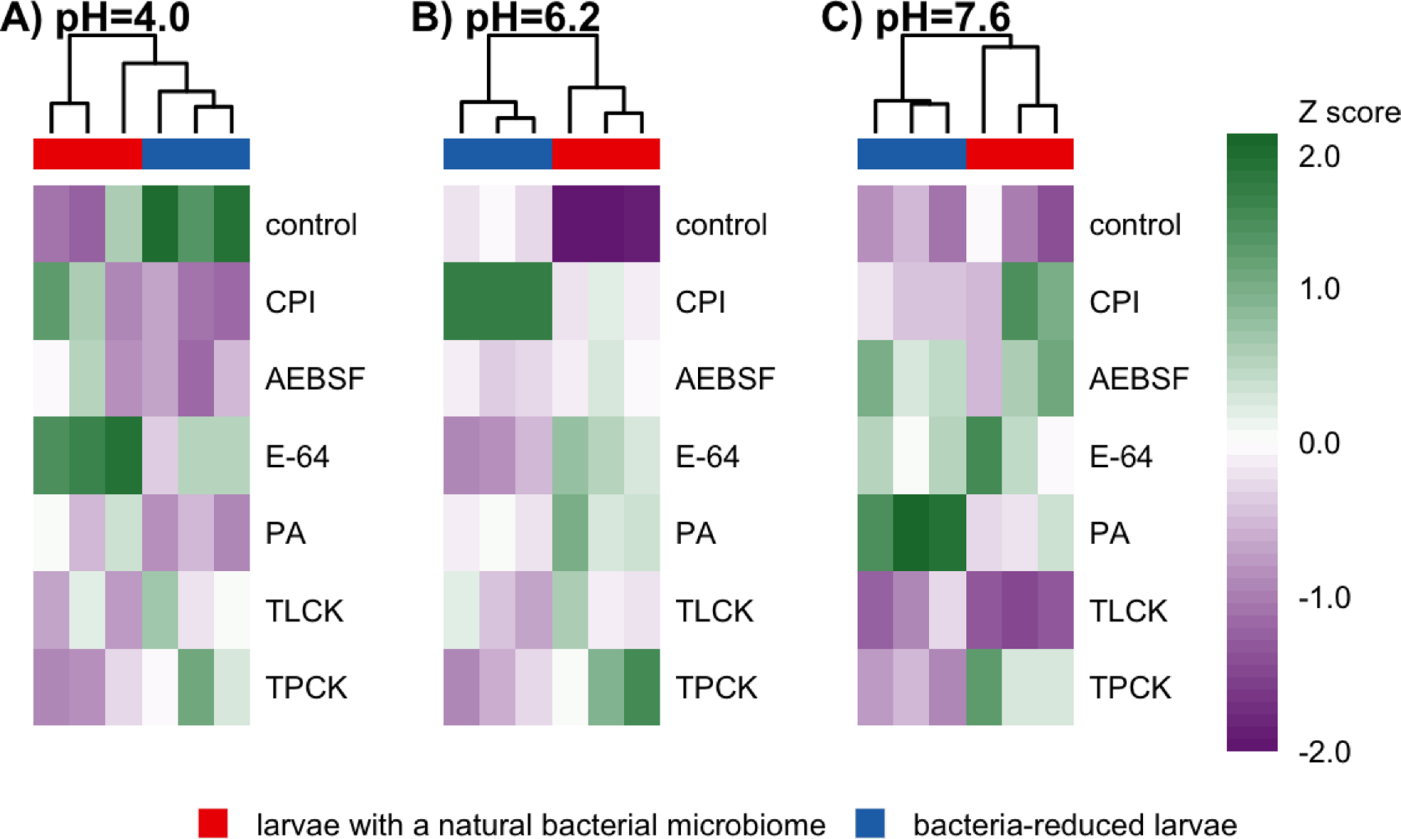
Hierarchical clustering analysis of protease activity patterns. Heatmaps show standardized median fluorescence intensity (Z-scores) across all experimental conditions at pH (**A**) 4.0, (**B**) 6.2, and (**C**) 7.6. Hierarchical clustering performed using complete linkage method shows grouping patterns between reduced bacterial community (red blocks) and larvae with a natural bacterial microbiome (blue block). Color intensity indicates Z-score values (scale: − 2.0 to + 2.0), with green indicating higher standardized activity and purple indicating lower standardized activity. Controls were insects with a natural microbiome (neither antibiotics nor protease inhibitor-treated) and larvae with reduced bacterial community (treated only with antibiotics). There were a total of 12 treatments, with 9 larvae (3 biological replicates of 3 pooled larvae each) for each. CLB larvae were collected from wheat fields in Słupia Wielka in 2021. CPI-cocktail of protease inhibitors, E-64-cysteine protease inhibitor, AEBSF-serine protease inhibitor, TLCK-trypsin protease inhibitor, TPCK-chymotrypsin protease inhibitor, PA-aspartyl protease inhibitor.

Hierarchical clustering analysis of protease inhibitor responses reveals distinct segregation patterns between larvae with a natural microbiome and specimens with reduced bacterial community across different pH conditions (Fig. 5). The study revealed several pronounced treatment-dependent effects. The existence of diverse and pH-dependent response patterns across various PIs suggests the presence of complex regulatory mechanisms. These mechanisms are further influenced by the presence of a natural microbiome and its antibiotic-induced reduction. These factors significantly alter proteolytic activity profiles in CLB larvae. The directionality and magnitude of these effects vary substantially by inhibitor type and environmental pH (Fig. 5).

### CLB-associated bacteria have a negative impact on the development of their insect host under laboratory conditions

**Fig. 6:**
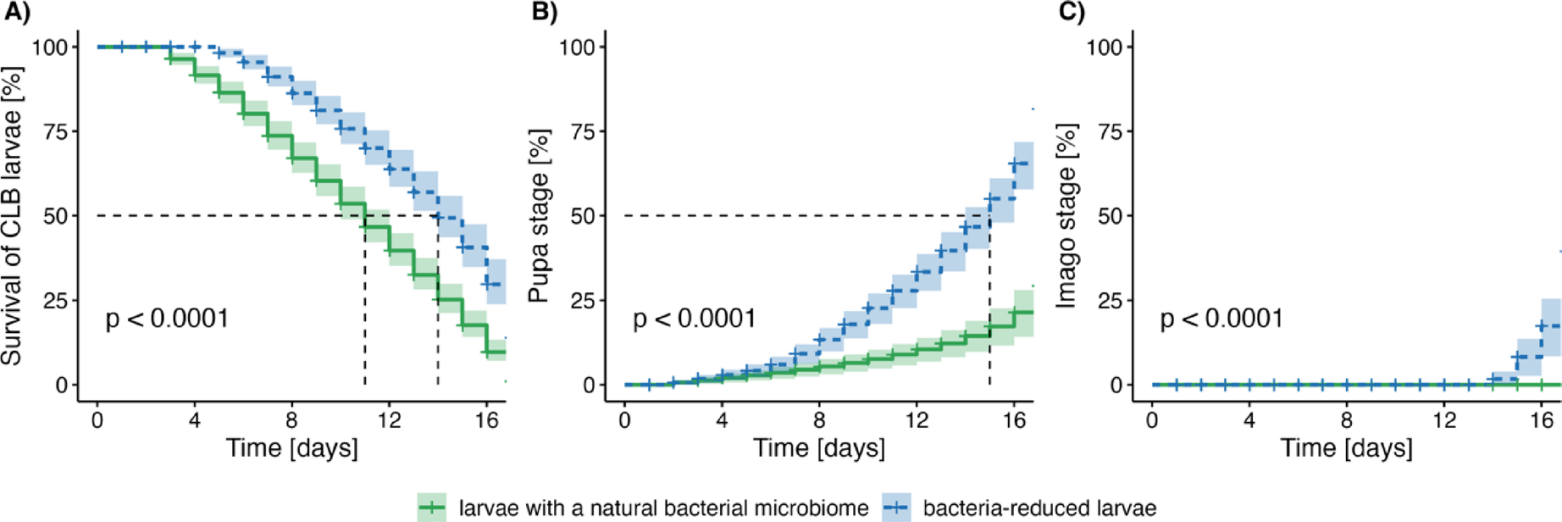
Developmental success of CLB larvae under different microbiome conditions. Kaplan–Meier curves depicting (**A**) the survival probability of CLB larvae over 17 days (**B**) cumulative proportion of CLB larvae that reached the pupa and (**C**) cumulative proportion reaching imago stage during 17 days of experiment. The green lines refer to larvae with a natural bacterial microbiome (control group), and the blue lines refer to larvae with reduced bacterial community. The shaded areas around the lines represent 95% confidence intervals. The dashed lines in panels A and B indicate the median time to event (M50) for larval mortality and pupation, respectively. Each treatment group contained a total of 30 larvae (3 replicated of 10 larvae each), which were collected from wheat fields in Słupia Wielka in 2022. Statistical comparisons between the natural and bacteria-reduced groups across all developmental endpoints were performed using log-rank tests and are indicated with p-values in each panel.

We assumed, that reduction of bacteria associated with CLB larvae has a negative impact on the acquisition of essential nutrients, which will result in a decrease in both the pupation and adult rate, but the laboratory assay showed higher survival rates in larvae with reduced bacterial community (that were consuming antibiotics), compared to those constituting the control (larvae with a natural microbiome) (Fig. 6A).

Interestingly, a significantly higher number of CLB larvae (~ 47% of the insects for this treatment) with reduced bacterial community reached the pupal stage than those with a natural microbiome, where only 10% of the insects for this treatment reached this stage (Fig. 6 B).

Moreover, ~ 27% of the insects with reduced bacterial community after the pupa stage, reached the imago stage in laboratory conditions. No imago stage was recorded for larvae with a natural microbiome (Fig. 6C).

There is a statistically significant difference in larval survival rate, the proportion of CLB larvae that reached the pupa and imago stage, between the group of insects with a natural and reduced bacterial microbiome (*p* < 0.001) (Fig. 6).

**Fig. 7:**
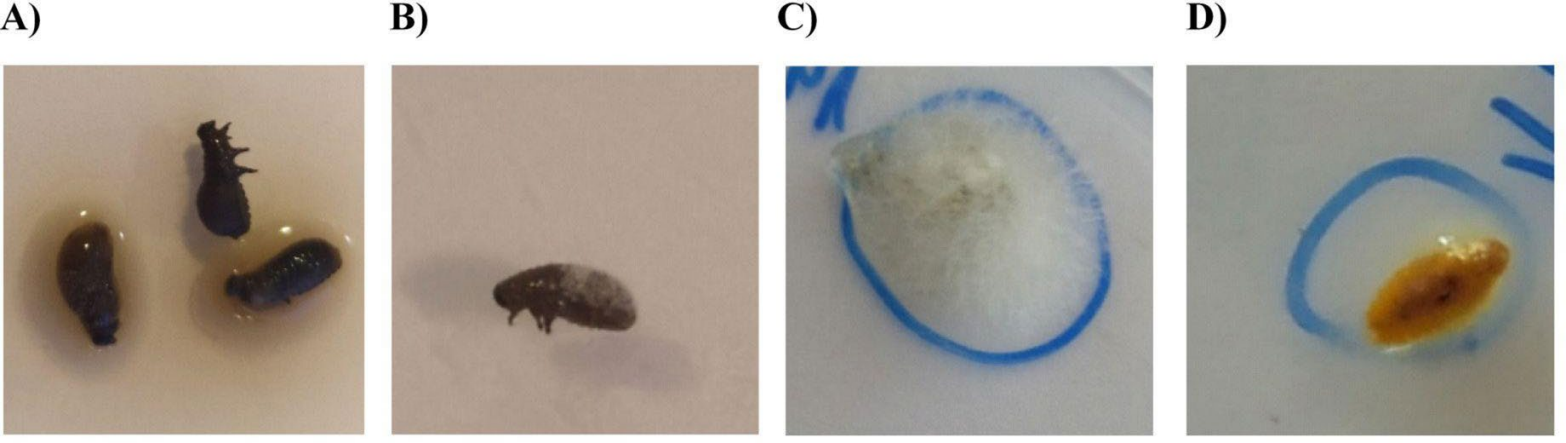
(**A**) CLB larvae with a natural microbiome surrounded by a gooey fluid. For the group of insects with reduced bacterial community, larvae (**B**), pupae (**C**) surrounded by mycelium and pupae (**D**) surrounded by gooey fluid were recorded.

During the experiment, most of the larvae constituting the control (insects with a natural microbiome) were observed to be black in colour and surrounded by a gooey fluid (Fig. 7A). In turn, some of the dead larvae in which the bacterial community was reduced were surrounded by mycelium (Fig. 7B). Mycelium was also observed for the pupal stage of larvae with reduced bacterial community (Fig. 7C), only one pupa was surrounded by gooey fluid (Fig. 7D).

## Discussion

Protease inhibitors are natural insecticides that interfere with insect digestive processes but insects have developed adaptive mechanisms to allow them to digest plant food containing PIs^3,14,15^. Insects’ adaptability to PIs includes, among others: i) increased expression of proteases insensitive to PIs, ii) activation of proteases that hydrolyze plant PIs, iii) up and/or down-regulation of existing proteases^29,30^. Many insects use a combination of the mentioned strategies^6^. In our previous research, we revealed that CLB larvae have developed adaptive mechanisms to PIs^15^. Increased proteolytic activity and the appearance of new protease isoforms have been reported in response to some PIs. In addition, we indicated that CLB possesses proteases from four mechanistic classes, which is beneficial for the insect in dealing with PIs^15^.

It is known that insect-associated bacteria can minimize the adverse effects of plant PIs on food digestion and nutrient uptake in two ways. Namely, these bacteria associated with the saliva of herbivorous insects can modulate plant response to the benefit of their insect host^28,31^, affecting the quality of the food consumed and, thus, in consequence, impacting the insect’s gut microbiome content^32^. Additionally, bacteria inhabiting the insect gut can support the digestive processes of the insect host by providing their own proteolytic enzymes^33,34^. Previously, we indicated that CLB-associated bacteria have a significant influence on the expression of a number of genes related to the defense response of wheat plants to damage caused by CLB larvae, including genes encoding PIs^14,28^. In this study, we have undertaken to analyse whether CLB-associated bacteria are able to support the digestive processes of CLB in response to PIs.

Although the CLB larvae after AB treatment were not completely free of bacteria, we assumed that this would disrupt the balance of the insect’s microbiome (dysbiosis), which would have negative impact on its development, immunity and its ability to cope with PIs. We revealed lower protease activities in larvae with reduced bacterial community after PI treatment (regardless of the type of PI used) compared to the activity recorded for insects with a natural microbiome exposed to the PI. This mainly involves cysteine and chymotrypsin proteases. This is particularly evident for a pH value of 6.2. Such a result suggests that CLB-associated bacteria participate in the adaptation of their insect host to PIs (bacterial-derived proteases may e.g., digest plant material, degrade PIs). The pH value for the digestive system of the CLB has not been determined. Based on previous studies, the highest protease activity for CLB was observed at an acidic pH in the range of 3–4^15^. It is known that pH is one of the factors influencing the activity of enzymes that break down food, and that insects have been shown to actively regulate the pH in their digestive system^35,36^. Moreover, different parts of an insect’s digestive system may have a specific pH range, and each region may exhibit differences in enzyme abundance^35,37^. The food consumed by the insect (alkaline or acidic) also has a significant impact on protease activity^35,37^. Interestingly, also insect-associated bacteria are able to affect oxygen concentration, pH, and redox state in the gut, which influences the fitness of the insect host^20,38^.

However, we reported higher survival of CLB larvae with reduced bacterial community compared to insects with a natural microbiome in response to PIs, in laboratory conditions. Literature data mainly indicate that bacteria provide amino acids to insects by digesting proteins from consumed plant material (via peptidase expression) or de novo amino acid biosynthesis^39^, and depriving the insect of bacteria has a negative effect on food digestion and insect growth and development. For instance, Berasategui et al.^40^ showed that bacteria associated with *Hylobius abietis* (Curculionidae, Coleoptera) by digesting diterpenes increased the nutritional properties of the food of their insect hosts and thus increased insect fitness. In turn, Erkosar et al.^41^ indicated that *Lactobacillus plantarum *^*WJL*^ associated with *Drosophila melanogaster* (Drosophilidae, Diptera) promoted intestinal peptidase expression, leading to increased intestinal proteolytic activity and, thus, dietary protein digestion and amino acid acquisition by *D. melanogaster*, which had a beneficial effect on growth and development.

In the present study, we also examined the effect of CLB-associated bacteria on the larva to pupa transition and reaching the adult stage. We hypothesized that reducing bacteria in the larval stage of CLB would have a negative impact on plant food digestion (on amino acids acquisition) and thus on larval survival, pupation, and reaching adulthood. CLB has four larval instars, a pupal stage, and an adult stage^42^. During pupation, the tissues of holometabolous insects, to which CLB belongs, undergo changes to allow the insect to reach the adult stage. Based on research by Chiang and Shelomi^43^, the digestive system for the subsequent developmental stages of CLB should have a similar morphological structure, but this needs to be confirmed by further research. Chiang and Shelomi^43^ demonstrated that the structure of the digestive tract of *Cassida circumdata* (Coleoptera, Chrysomelidae), in which the adult and larva have the same feeding habits (feed on the leaves, leaving short holes), remains relatively the same for larval, pupal and adult stages. However, the gut microbiome for *C. circumdata* was not examined. CLB and *C. circumdata* belong to the same family (Chrysomelidae) and the larval and adult stages of CLB also have the same feeding habits (they feed on leaves), which is why we referred to Chiang’s and Shelomi^43^ research. In previous research, we indicated that larva and adult stages of CLB differ in terms of the structure of the bacterial microbiome, which depends highly on the host plant species they fed on^44^. In the present study, we indicated that the reduction of bacteria in CLB larvae significantly impacted the survival rate of larvae, pupation and the emergence of adults in laboratory conditions. Contrary to our assumption, almost half of larvae with reduced bacterial community reached the pupa and 27% of them the adult stage, in contrast to larvae with a natural microbiome (control), where none of the larvae reached the adult stage (only 10% of them reached the pupal stage). It was previously found that reducing the bacterial community has a negative effect on the development of the insect. For instance, in *Aedes aegypti* (Culicidae, Diptera) mosquito larvae deprived of bacteria, downregulation of peptidase genes (including serine hydrolase, trypsin, ß-aspartyl peptidase, metallopeptidase) was noted, which affected the acquisition and assimilation of nutrients, and thus the larvae did not grow or molt^45^. Xiaowen et al.^46^ indicated that tetracycline treatment disrupted the gut bacterial community in larvae of *Apis cerana* (the Asian honeybee, Apidae, Hymenoptera) and led to a significant decrease in both the pupation rate and emergence rate. Chouaia et al.^47^ recorded that larvae of *Anopheles stephensi* (Culicidae, Diptera) treated with the antibiotic (rifampicin) showed a delay in the development and an asynchrony in the appearance of later instars. Liu et al.^20^ indicated that bacteria inhabiting the gut of *Dendroctonus valens* LeConte (the red turpentine beetle (RTB), Curculionidae, Coleoptera) are essential for the proper development of RTB larvae by regulating D-glucose transport. RTB larvae deprived of bacteria by antibiotics exhibited significantly reduced growth compared to insects with a natural gut microbiome. But recently, the positive effect of reducing bacteria on insect survival was also reported. Namely, Garrigós et al.^48^ found that exposure of *Culex pipiens* (Culicidae, Diptera) mosquito larvae to antibiotics significantly increased the survival rate of adult females that received a control diet (sugar solution).

We wondered why a group of larvae with a natural microbiome had a higher mortality rate in response to PIs and failure to reach the adult stage compared to insects with reduced bacterial community. According to Zhang et al.^4^ this may be due to the fact that bacteria support the insect when the plant’s defenses are relatively weak. When the plant’s response is strong or when the insect is in poor condition, the bacteria often turn pathogenic, resulting in increased insect mortality. While studying the impact of bacteria on the development stages of the CLB, we noticed that dead larvae were surrounded by gooey fluid (probably bacteria). We assume that the balance in the insect’s intestinal community was disrupted, leading to the death of the larvae.

The effect of bacterial depletion on the development of the insect probably also depends on the length of exposure of the insect to antibiotics, the type of antibiotics, and their dose. The development stage of the insect is also important in dealing with PIs. Zhu-Salzman et al.^29^ demonstrated that feeding inhibition and growth retardation caused by dietary soyacystatin N (scN, the soybean cysteine protease inhibitor) only occurred during the earlier developmental stages (the 1st, 2nd, and 3rd instars) of the *Callosobruchus maculatus* (Coleoptera, Chrysomelidae). The 4th instar larvae were able to recover their normal feeding and growth in the presence of scN in the diet. However, it should be emphasized and taken into account that studies of the effect of PIs and bacteria on protease activity and the development of the insect, including those presented in the article, were performed under laboratory conditions, which do not reflect field conditions. Changing temperature, humidity, and interactions with the environment can affect the insect microbiome content and influence insect development.

## Conclusion

Our results provide confirmation that the presence of insect-associated bacteria contributes to the CLB larvae’s adaptation to PIs. On the other hand, it has been shown that under laboratory conditions, the presence of a natural microbiome had an adverse effect on the development of CLB, i.e., reducing larval survival and progression to subsequent developmental stages. There are still a lot of unknowns about the relationship between the insect and bacteria in beetles. The microbial community is often crucial for the nutritional status and survival of their insect hosts^32^. Any alteration in the insect’s microbiome can significantly affect its biology, fitness, population dynamics and adaptability to environmental changes or new host plants^32^. A better knowledge of CLB and its associated communities (holobiont), including bacterial community, may contribute to the improvement of integrated pest management tools against cereal pests in the future. The diverse roles and effects of symbionts on insect traits have now gained new perspectives for biotechnological exploration. Technological innovations, such as the use of RNA interference (RNAi) in plant protection using symbiotic microbes as carriers, appear promising^16,49^.

## Materials and methods

### Materials

Inhibitors: a cocktail of protease inhibitors (CPI, cat. no. P9599), pepstatin A (PA, cat. no. P5313), trans-epoxysuccinyl-L-leucylamido-4 guanidino butane (E-64, cat. no. E3132), 4-(2-aminoethyl) benzenesulfonyl fluoride hydrochloride (AEBSF, cat. no. A8456), Nα-tosyl-L-lysine chloromethyl ketone hydrochloride (TLCK, cat. no. T4376), N-p-tosyl-L-phenylalanine chloromethyl ketone (TPCK, cat. no. T4376), a Protease Fluorescent Detection Kit (cat. no. PF0100) and chlortetracycline (cat. no. C4881) were purchased from Merck (Poznań, Poland). Streptomycin sulfate (cat. no. S6501), neomycin sulfate (cat. no. N1876) were purchased from Epro (Puck, Poland; producer BioShop Life Science). Qubit™ Protein Assay Kit (cat. no. Q33212) and Maxima First Strand cDNA Synthesis Kit (cat. no. K1672) were purchased from Thermo Fisher Scientific (Warsaw, Poland). The Total RNA Mini Concentrator kit (cat. no. 031-100C) was purchased from A and A Biotechnology (Gdańsk, Poland). 2X QX200 EvaGreen Supermix were purchased from BioRad (Warsaw, Poland).

### Determination of antibiotic concentrations to obtain CLB larvae with reduced bacterial community

CLB larvae (3rd instar) were collected from a field of winter wheat in Słupia Wielka (52°13′02″N 17°13′04″E) in 2021 and transported to the laboratory (conditions 20 ± 2 °C, without additional lighting) of the Institute of Plant Protection – National Research Institute, Poznań, Poland. Immediately after collection, the insects were starved for 24 h. Next larvae fed for 48 h on wheat leaves (previously disinfected in 70% ethanol, rinsed twice in sterile Milli-Q water, and placed on a 1% agarose medium (Petri dishes) to retain moisture) completely covered by a mixture of antibiotics (AB) by immersing the leaves in the solution of AB (three following antibiotics: neomycin sulfate (0.02%), chlortetracycline (0.1%) and streptomycin sulfate (0.006%) were suspended together in 50 mL of ultrapure Milli-Q water) at a concentration of 1 (AB( +) × 1, initial concentration: neomycin sulfate (0.02%), chlortetracycline (0.1%) and streptomycin sulfate (0.006%)), 10 (AB( +)/10, neomycin sulfate (0.002%), chlortetracycline (0.01%) and streptomycin sulfate (0.0006%)) and 100 (AB( +)/100, neomycin sulfate (0.0002%), chlortetracycline (0.001%) and streptomycin sulfate (0.00006%)) times dilution to reduce bacteria present in CLB larvae. To the AB solution, 5 µl of Triton X-100 was added to reduce the surface tension of the liquid. Four pieces of wheat leaf (measuring 1 × 4 cm) and 10 CLB larvae were placed on each Petri dish. Insects were treated with AB according to Chung et al.^31^ with some modification. The control was AB-untreated larvae (natural microbiome), which were also starved for 24 h before being applied to the wheat leaf. After 48 h of feeding on leaves, the insects were placed in 70% ethanol and stored at − 20 °C. There were 10 larvae for each treatment (a total of 40 larvae were used in the experiment). Before RNA isolation, CLB larvae underwent surface sterilization by washing them 3 times with sterile water. RNA from each individual larva was isolated using a Total RNA Mini Concentrator kit, and then 1 μg of RNA was transcribed into cDNA with a DNase step using a Maxima First Strand cDNA Synthesis Kit. cDNA samples diluted 1,500 times were used for digital droplet PCR (ddPCR) reaction. The ddPCR assay was performed in 20 µl of reaction mixture containing 10 µl of 2X QX200 EvaGreen Supermix, 0.2 µl of a mixture of forward and reverse primers (see Supplementary Table S3 online) at a concentration of 10 µM, 1 µl of cDNA, and supplemented with water to the final volume. The specificity of the primers was verified using the nucleotide blast website (https://blast.ncbi.nlm.nih.gov/Blast.cgi). Based on the results obtained, the possibility of obtaining PCR products from mitochondrial or chloroplast RNA was ruled out. Negative controls were controls of the extraction process (NTC) and samples to which water was added instead of cDNA (C(-)). The positive control was the plasmid containing the 16S rRNA gene product obtained by using the primers (listed in Supplementary Table S3). The reaction was carried out according to the manufacturer’s recommendations using the Bio-Rad ddPCR. The fluorescence signal in all samples tested was read using a QX200 Droplet Reader, and the absolute copy number of bacterial cDNA was calculated using QuantaSoft software, which uses the Poisson formula.

### Assessing the impact of bacteria on the survival of larvae in response to protease inhibitors

CLB larvae (3rd instar) collected from wheat fields in Słupia Wielka in 2021 and 2022 and from Pętkowo (52°12′32″N 17°15′17″E) in 2022 were transferred to the laboratory (conditions 20 ± 2 °C, without additional lighting) of the Institute of Plant Protection-National Research Institute, Poznań, Poland. Firstly, larvae were starved for 24 h. Next, in some CLB larvae, the number of bacteria was reduced using an initial concentration of AB (AB( +) × 1, neomycin sulfate (0.02%), chlortetracycline (0.1%) and streptomycin sulfate (0.006%)) according to the procedure described in section ,,Determination of antibiotic concentrations to obtain CLB larvae with reduced bacterial community’’ to test whether the presence of bacteria affects the survival of PI-treated larvae.

Next, CLB larvae with a natural (AB-untreated) and reduced bacterial microbiome (AB-treated) were exposed to the following PIs, separately: E-64 (cysteine protease inhibitor), PA (aspartyl protease inhibitor), AEBSF (serine protease inhibitor), TLCK (trypsin protease inhibitor), TPCK (chymotrypsin protease inhibitor) and cocktail of protease inhibitors (CPI, the cocktail contains a broad specificity of inhibitory properties including serine, cysteine, aspartic and metalloproteases, and aminopeptidases). Five micrograms of PI were placed with a pipette + on the previously disinfected wheat leaf (rinsed once in 70% ethanol and twice in autoclaved distilled water), and placed on a plate of 1% agar to keep the leaf moist. The controls were larvae treated with the AB mixture alone and untreated larvae (with neither a mixture of AB nor the PI). Four pieces of wheat leaf (measuring 1 × 4 cm) and 20 CLB larvae were placed on each Petri dish. Evaluation of larval survival was performed 24 and 48 h after larvae were exposed to PIs. Within 48 h, the larvae had eaten almost the entire surface of the leaves. There were a total of 12 treatments, with 20 larvae for each. A total of 720 larvae collected from two locations were used in the experiment.

CLB larvae (3rd instar) collected from wheat fields in Słupia Wielka in 2021 were used for the next step of the experiment, i.e., Measurement of protease activity associated with CLB larvae’’ to test whether bacteria participate in the adaptation of CLB larvae to PIs through involvement in the digestive system of the insect host. Therefore, after 72 h of feeding on wheat leaves covered by PIs, larvae were placed in 0.1 M Tris buffer (pH 7.5) containing 20% glycerol and stored at—20 °C until the protein was isolated.

### Measurement of protease activity associated with CLB larvae

#### Protein isolation

Because the larvae were too small for intestinal isolation and there was a likelihood of sample contamination during intestinal isolation, we used whole-larval preparations for all enzyme assays. Prior to protein isolation, the CLB larvae were subjected to surface sterilization by washing them 3 times with sterile water to remove any residual fecal coat (which in field conditions protects them from the sun, among other things) in order to eliminate contamination of the larval homogenate. Larvae were homogenized using a sterile arrow in 100 µl of 0.1 M Tris buffer (pH 7.5) containing 20% glycerol. The sample consisted of 3 larvae (homogenized together), with a total of 3 samples per treatment (total of 9 larvae). Only those larvae that were alive after the PI treatment step were used for protein isolation. Preparations were centrifuged at 16,000 × g for 5 min at 4 °C. The supernatant was transferred to fresh tubes and again centrifuged at 16,000 × g for 2 min at 4 °C. Cleared supernatant was collected and stored at—20 °C until measurements of total proteases’ activity associated with CLB larvae were performed.

#### Determination of protein concentration

The protein concentration was determined by using the Qubit™ Protein Assay Kit and Qubit 4 Fluorometer.

#### Spectrophotometric measurement of protease activity

The activity of proteases associated with CLB larvae was determined by using the Protease Fluorescent Detection Kit. Twenty microliters of incubation buffer (25 mM sodium acetate–phosphate–glycine buffer at the desired pH values: 4.0, 6.2, 7.6) and 20 μl of fluorescein isothiocyanate (FITC)-casein substrate were added to 10 μl (containing 10 µg protein) of each sample tested. The blank (negative control) was a sample to which water was added, while to the positive sample (checking whether the process of substrate decomposition is taking place), instead of protein extract, enzyme trypsin was added.

The reaction was carried out at 37 °C for 19 h in the dark. After this time, the reaction was stopped by adding a cold trichloroacetic acid solution and kept at 37 °C for 30 min in the dark. Next, samples were centrifuged for 10 min at 10,000 × g to remove the undigested substrate and the smaller, acid-soluble fragments. Ten microliters of supernatant were added to 1.0 ml of assay buffer (500 mM Tris buffer at pH 8.5). Two hundred microliters of the supernatant solution and assay buffer were transferred to the well of a black 96-well plate. Two reactions were performed for each biological sample. Additionally, each reaction was applied twice to the plate for reading (two technical repetitions).

The fluorescence measurement of the samples was made at an excitation wavelength of 485 nm and an emission wavelength of 535 nm using a DTX 880 Multimode Detector (Beckman Coulter Life Science).

### Pupation and adult stage rate assessment

CLB larvae (3rd instar) collected from wheat fields in Słupia Wielka in 2022 were used to test the effect of bacteria on the development of this insect, i.e., reaching the pupa stage and then the adult stage. The experiment was conducted in a laboratory (20 ± 2 °C, without additional lighting). Some of the larvae were treated with an initial concentration of AB (AB( +) × 1, neomycin sulfate (0.02%), chlortetracycline (0.1%) and streptomycin sulfate (0.006%)) according to the procedure described in section,Determination of antibiotic concentrations to obtain CLB larvae with reduced bacterial community’’. Controls were AB-untreated insects (with a natural bacterial microbiome). After 48 h of CLB larvae feeding on leaves covered with AB, the leaves were replaced with new pieces of wheat leaves (previously disinfected according to the procedure described above), which were replenished approximately every 2–3 day as needed. There were three repetitions of 10 insects per treatment (a total of 60 larvae per experiment). Over a period of 17 days, the survival rate of larvae, the formation of pupae and the emergence of adults were assessed.

### Statistical analysis

Fluorescence intensities were calculated using blanks as a benchmark. Data were pre-processed to filter out outliers using the inner-quartile range (IQR) method. For each group, samples with fluorescence values differing from the median by more than 1.5 IQR were excluded from analysis. Next, for the analysis of protease activity, a generalized linear model with a negative binomial distribution (GLM NB) was fitted using the R package MASS^50^. It modelled median fluorescence intensity (MFI). The MFI values served as the dependent variable, which exhibited overdispersion typical of count-like data, making the negative binomial distribution appropriate for modeling. The model included the type of PI, antibiotic treatment status (AB), and pH as fixed effects along with all possible interactions between these factors. Model selection was performed by comparing models using the R package performance^51^, which provided comprehensive model comparison metrics including AIC, BIC, and likelihood ratio tests. The final model formula was: MFI ~ PI × AB × pH. The influence of variables was determined using Analysis of Deviance with chi-square tests^52^. Post-hoc analysis of protease activity data was performed using estimated marginal means (EMMs) calculated with the R package emmeans. Planned contrasts were constructed to compare larvae with reduced bacterial community versus larvae with a natural bacterial microbiome for each combination of PI type and pH level. The Wald tests with asymptotic normal distribution (z-tests) was used for these comparisons. The heatmap, which was prepared using hierarchical clustering with the method complete^53^ for the fluorescence intensity of the considered conditions, is also presented.

In survival analysis, Kaplan–Meier curves were constructed using the R package survival^53^. Log-rank tests were used to compare survival between treatment groups. Cox proportional hazards models were fitted when appropriate, with hazard ratios and 95% confidence intervals reported.

All statistical analyses were performed in R version 4.4.1.

## Supplementary Information

Below is the link to the electronic supplementary material.

Supplementary Material 1

## Data Availability

Data is provided within the manuscript or supplementary information files.
