# Supplementary material for: Bacteria associated with the cereal leaf beetle act as the insect’s allies in adapting to protease inhibitors, but impair its development in laboratory condition
Source: Sci Rep. 2025 Nov 14;15:39944. doi: 10.1038/s41598-025-23674-9 (PMC12618534; doi:10.1038/s41598-025-23674-9)
Supplement: Supplementary file 1 — Supplementary Material 1 [file 41598_2025_23674_MOESM1_ESM.docx]

**Supporting Information**

**Bacteria associated with the cereal leaf beetle act as the insect's allies in adapting to protease inhibitors, but impair its development in laboratory condition**

Beata Wielkopolan,^a^ Alicja Szabelska-Beręsewicz,^b^ Aleksandra Obrępalska-Stęplowska^c*^

^a^ Department of Monitoring and Signaling of Agrophages, Institute of Plant Protection–National Research Institute, Poznan, Poland

^b^ Department of Mathematical and Statistical Methods, Poznan University of Life Sciences, Poznan, Poland

^c^ Department of Molecular Biology and Biotechnology, Institute of Plant Protection–National Research Institute, Poznan, Poland

*Correspondence to: Aleksandra Obrępalska-Stęplowska, Department of Molecular Biology and Biotechnology, Institute of Plant Protection–National Research Institute, Poznan, Poland. E-mail: [olaob@o2.pl](mailto:olaob@o2.pl)


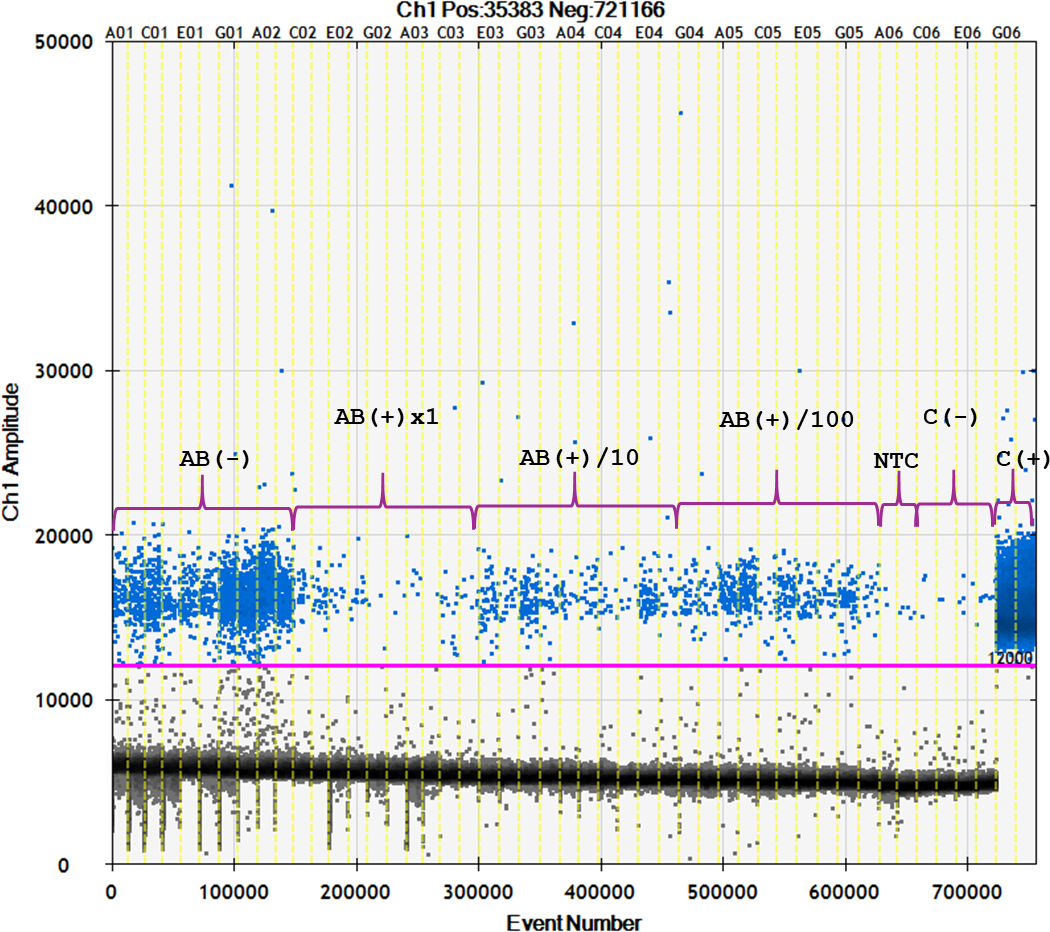


**Fig. S1.** The expression level of the 16S rRNA gene of bacteria associated with CLB larvae. The expression level of the 16S rRNA gene by ddPCR was determined in larvae with a natural microbiome (AB(-)) and in larvae in which the bacterial content was reduced by using the initial antibiotics concentration (AB(+)x1) and their 10-fold (AB(+)/10) and 100-fold (AB(+)/100) dilutions. There were 10 CLB larvae for each treatment. Negative controls were controls of the extraction process (NTC) and samples to which water was added instead of cDNA (C(-)). The positive control was the plasmid containing the 16S rRNA gene product. On the Y axis – the amplitude of fluorescence of positive (blue) and negative (gray) droplets and on the X axis – the number of events. Diagram generated using QuantaSoft software (Bio-Rad).

**Table S1.** The estimated fold change in mean fluorescence intensity value protease inhibitor (PI)-treated larvae vs control for each pH value considered (4.0, 6.2, 7.6) is reported. The data from PI-treated insects with a natural and reduced bacterial microbiome were compared to the corresponding control, i.e., neither antibiotics nor PI-treated and treated only with antibiotics, respectively. The experiment involved a total of 12 treatments, with 20 larvae for each. The CLB larvae were collected from wheat fields in Słupia Wielka in 2021.

CPI-cocktail of protease inhibitors, AEBSF-serine protease inhibitor, E-64-cysteine protease inhibitor, PA-aspartyl protease inhibitor, TLCK-trypsin protease inhibitor, TPCK-chymotrypsin protease inhibitor. Significant codes for p-value: *p < 0.05, **p < 0.01, ***p < 0.001.

|  |  | larvae with a natural microbiome | | bacteria-reduced larvae | |
| --- | --- | --- | --- | --- | --- |
| PI | pH value | fold change | p-value | fold change | p-value |
| CPI | 4 | 1.2900 | 0.1081 | 0.6311 | 0.0037** |
| AEBSF | 4 | 1.1925 | 0.2666 | 0.6532 | 0.0072** |
| E-64 | 4 | 1.4674 | 0.0155* | 0.7575 | 0.0798 |
| PA | 4 | 1.1454 | 0.3918 | 0.6441 | 0.0055** |
| TLCK | 4 | 1.1174 | 0.4838 | 0.8084 | 0.1797 |
| TPCK | 4 | 1.0294 | 0.8550 | 0.7826 | 0.1220 |
| CPI | 6.2 | 2.6231 | <0.0001*** | 1.5871 | 0.0036** |
| AEBSF | 6.2 | 2.7020 | <0.0001*** | 0.9756 | 0.8760 |
| E-64 | 6.2 | 3.1138 | <0.0001*** | 0.8517 | 0.3111 |
| PA | 6.2 | 3.1443 | <0.0001*** | 1.0270 | 0.8663 |
| TLCK | 6.2 | 2.7393 | <0.0001*** | 0.9597 | 0.7950 |
| TPCK | 6.2 | 3.3686 | <0.0001*** | 0.9024 | 0.5168 |
| CPI | 7.6 | 1.5218 | 0.0081** | 1.1823 | 0.2907 |
| AEBSF | 7.6 | 1.4449 | 0.0202* | 1.5614 | 0.0049** |
| E-64 | 7.6 | 1.4505 | 0.0190* | 1.4772 | 0.0138* |
| PA | 7.6 | 1.2869 | 0.1116 | 2.0323 | <0.0001*** |
| TLCK | 7.6 | 0.8562 | 0.3272 | 1.0300 | 0.8521 |
| TPCK | 7.6 | 1.4658 | 0.0158* | 1.0228 | 0.8871 |

**Table S2.** Pairwise comparisons of total activity of proteases of larvae with reduced bacterial community vs insects with a natural microbiome, depending on the type of protease inhibitor (CPI, AEBSF, E-64, PA, TLCK, TPCK) and pH value (4.0, 6.2, 7.6). Controls were insects with a natural microbiome (neither antibiotics nor protease inhibitor treated) and larvae with reduced bacterial community (treated only with antibiotics). The experiment involved a total of 12 treatments, with 20 larvae for each. The CLB larvae were collected from wheat fields in Słupia Wielka in 2021. Results were demonstrated as fold change of mean fluorescence intensity value for each comparison. Values above 1 indicate increased MFI in bacteria-reduced larvae. CPI-cocktail of protease inhibitors, AEBSF-serine protease inhibitor, E-64-cysteine protease inhibitor, PA-aspartyl protease inhibitor, TLCK-trypsin protease inhibitor, TPCK-chymotrypsin protease inhibitor. Significant codes for p-value: *p < 0.05, **p < 0.01, ***p < 0.001.

|  | pH = 4.0 | | pH = 6.2 | | pH = 7.6 | |
| --- | --- | --- | --- | --- | --- | --- |
|  | fold change | p-value | fold change | p-value | fold change | p-value |
| Control | 1.3201 | 0.0798 | 1.7199 | 0.0006*** | 0.7400 | 0.0575 |
| CPI | 0.6457 | 0.0058** | 1.0406 | 0.8017 | 0.5749 | 0.0005*** |
| AEBSF | 0.7230 | 0.0407* | 0.6210 | 0.0026** | 0.7997 | 0.1584 |
| E-64 | 0.6815 | 0.0155* | 0.4704 | <0.0001*** | 0.7537 | 0.0744 |
| PA | 0.7423 | 0.0601 | 0.5618 | 0.0003*** | 1.1687 | 0.3253 |
| TLCK | 0.9551 | 0.7718 | 0.6025 | 0.0014** | 0.8903 | 0.4633 |
| TPCK | 1.0036 | 0.9818 | 0.4607 | <0.0001*** | 0.5163 | <0.0001*** |

**Table S3.** Primer sequences used for dd-PCR analysis

| Gene | Prime sequence | Amplicon length [bp] | Annealing temperature [°C] | References |
| --- | --- | --- | --- | --- |
| 16S rRNA | F: 16S06 (GCTACACACGTGCTACAATG)  R: pH2 (AAGGAGGTGATCCAGCCGCA) | 280 | 60 | ^1^  ^2^ |

1. Gerischer U. Acinetobacter: *Molecular Biology*. 358 (2008)

2. Edwards, U., Rogall, T.Blöcker, H., Emde, M. & Böttger EC. Isolation and direct complete nucleotide determination of entire genes. Characterization of a gene coding for 16S ribosomal RNA. *Nucleic Acids Res*. **17**, 7843–53 (1989)
